# Strontium Functionalized in Biomaterials for Bone Tissue Engineering: A Prominent Role in Osteoimmunomodulation

**DOI:** 10.3389/fbioe.2022.928799

**Published:** 2022-07-06

**Authors:** Jiaqian You, Yidi Zhang, Yanmin Zhou

**Affiliations:** Department of Oral Implantology, Jilin Provincial Key Laboratory of Tooth Development and Bone Remodeling, Hospital of Stomatology, Jilin University, Changchun, China

**Keywords:** strontium, bone regeneration, macrophage, immunomodulatory, biomaterials

## Abstract

With the development of bone tissue engineering bio-scaffold materials by adding metallic ions to improve bone healing have been extensively explored in the past decades. Strontium a non-radioactive element, as an essential osteophilic trace element for the human body, has received widespread attention in the medical field due to its superior biological properties of inhibiting bone resorption and promoting osteogenesis. As the concept of osteoimmunology developed, the design of orthopedic biomaterials has gradually shifted from “immune-friendly” to “immunomodulatory” with the aim of promoting bone healing by modulating the immune microenvironment through implanted biomaterials. The process of bone healing can be regarded as an immune-induced procedure in which immune cells can target the effector cells such as macrophages, neutrophils, osteocytes, and osteoprogenitor cells through paracrine mechanisms, affecting pathological alveolar bone resorption and physiological bone regeneration. As a kind of crucial immune cell, macrophages play a critical role in the early period of wound repair and host defense after biomaterial implantation. Despite Sr-doped biomaterials being increasingly investigated, how extracellular Sr^2+^ guides the organism toward favorable osteogenesis by modulating macrophages in the bone tissue microenvironment has rarely been studied. This review focuses on recent knowledge that the trace element Sr regulates bone regeneration mechanisms through the regulation of macrophage polarization, which is significant for the future development of Sr-doped bone repair materials. We will also summarize the primary mechanism of Sr^2+^ in bone, including calcium-sensing receptor (CaSR) and osteogenesis-related signaling pathways.

## 1 Introduction

With the aggravation of the aging population and the increased incidence of fractures, the need for bone reconstruction is gradually increasing (Ray et al., 2018). Although autologous bone grafting is still the gold standard for restoring bone defects, this approach has the disadvantages of limited sources of donor bone tissue and the risk of infection in the donor site, which limits its widespread application in clinical practice. At the same time, there are some problems in allogeneic bone grafts, such as immunological reactions when implanted into human tissues. Therefore, developing an ideal bone substitute through BTE has become an urgent clinical problem to solve. In the past decade, the poor mechanical properties of some biomaterials hinder further development, even though the synthetic bone grafts based on ceramics and bioactive glass have been widely explored. So the way of adding metallic elements such as strontium, silicon, and magnesium iron into repair materials to improve their mechanical properties began to be proposed (Yang et al., 2021; Zhong et al., 2022). At present, a series of Sr-doped biomaterials have been proved to be an effective therapeutic tool for promoting bone regeneration.

Sr^2+^ is an alkali-earth metal cation that has been linked to the control of bone metabolism as one of the essential trace elements in the human body. Sr^2+^ has been shown to possess “dual regulation,” in which it stimulates osteoblasts to create new bone matrix while suppressing osteoclast activity and reducing bone resorption at the same time (No et al., 2017; Jimenez et al., 2019; Wang et al., 2019; Zeng et al., 2020; Geng et al., 2021). Sr^2+^ is active and exists widely in nature in the form of a combined state and is located on the fourth cycle and IIA group in the periodic table of chemical elements. On the other hand, Sr^2+^ usually exists in human bones that 99% of strontium (36–140 mg/kg) is precipitated in the femur, lumbar spine, and iliac bone, while the remaining 0.7% is found in extracellular fluid. Since Sr^2+^ is homologous with Ca^2+^ in chemical structure and chemical properties, it can substitute the Ca^2+^ positions of hydroxyapatite, leading Sr^2+^ with a substantial bone-seeking property (Cheshmedzhieva et al., 2021). Sr^2+^ mainly penetrates into bone tissue in two ways: 1) Most Sr^2+^ penetrates the lattice surface of bone mineral crystals through exchange with Ca^2+^. 2) Another small part of Sr^2+^ replaces Ca^2+^ of hydroxyapatite crystals in bone (Wu et al., 2013). At present, Sr^2+^ has been successfully applied in the osteoporosis therapy, among which strontium ranelate has been widely used in clinical practice and has shown an ideal therapeutic effect in the treatment of osteoporosis patients (Neves et al., 2017; Marx et al., 2020). Numerous researches have shown that a range of Sr^2+^-doped biomaterials, such as Sr^2+^-doped bone cement, Sr^2+^-doped bioactive glasses and Sr^2+^-composite bioactive coatings, can further accelerate bone formation around the bone defect (Kargozar et al., 2019; Naruphontjirakul et al., 2021).

According to the latest research reports, the immune response triggered during bone tissue regeneration shows that bone regeneration is closely related to the immune microenvironment surrounding the implanted biomaterials (Clézardin et al., 2021; Qiao et al., 2021; Chu et al., 2022). Advances in BTE have derived a consensus that the physicochemical characteristics of biomaterials can alter the microenvironment at the implantation site by affecting the inflammatory response (Li et al., 2017). The design of bone repair materials has gradually shifted towards biomaterials-mediated osteogenesis, in which stem cells directly modulate osteogenesis or vascularization to achieve the desired bone regeneration. Biomaterials will stimulate the innate immune system immediately when implanted in the body, recruiting immune cells to the surface of the biomaterials and triggering an immune response and local tissue inflammation in the host (Trindade et al., 2016; Sheikh et al., 2017). During this process, monocytes in the host immune system adhere to the surface of the implanted biomaterial and differentiate into macrophages. Under different microenvironmental conditions, macrophages that play a dynamic and superior plastic role can be polarized into pro-inflammatory phenotype (M1) and anti-inflammatory phenotype (M2). As the immune cells that first appear in the early inflammatory sites, macrophages have a wide range of associations with other cells via the secretion of various cytokines (Spiller and Koh, 2017). Cytokines secreted by macrophages recruit other immune cells to trigger the immune-inflammatory response and subsequent bone formation (Chu et al., 2019). Macrophage response in the implantation not only determines the effect of the initial inflammatory response but also the effect of ultimate bone formation. Therefore, the development of bone repair biomaterials should not only focus on the direct regulation of osteoblasts but also emphasize the modulation of the local inflammatory response. Only in that way can a suitable microenvironment of bone immune tissue would be formed. This review will summarize the relationship between trace element strontium and osteogenesis and focus on the mechanism of Sr^2+^ promotes osseointegration by inducing macrophage polarization to provide a reference for the application of Sr^2+^ in biomaterials.

## 2 Pharmacological Effect of Strontium on Bone Cells

### 2.1 Effects of Sr on Mesenchymal Stem Cells

**FIGURE 1:**
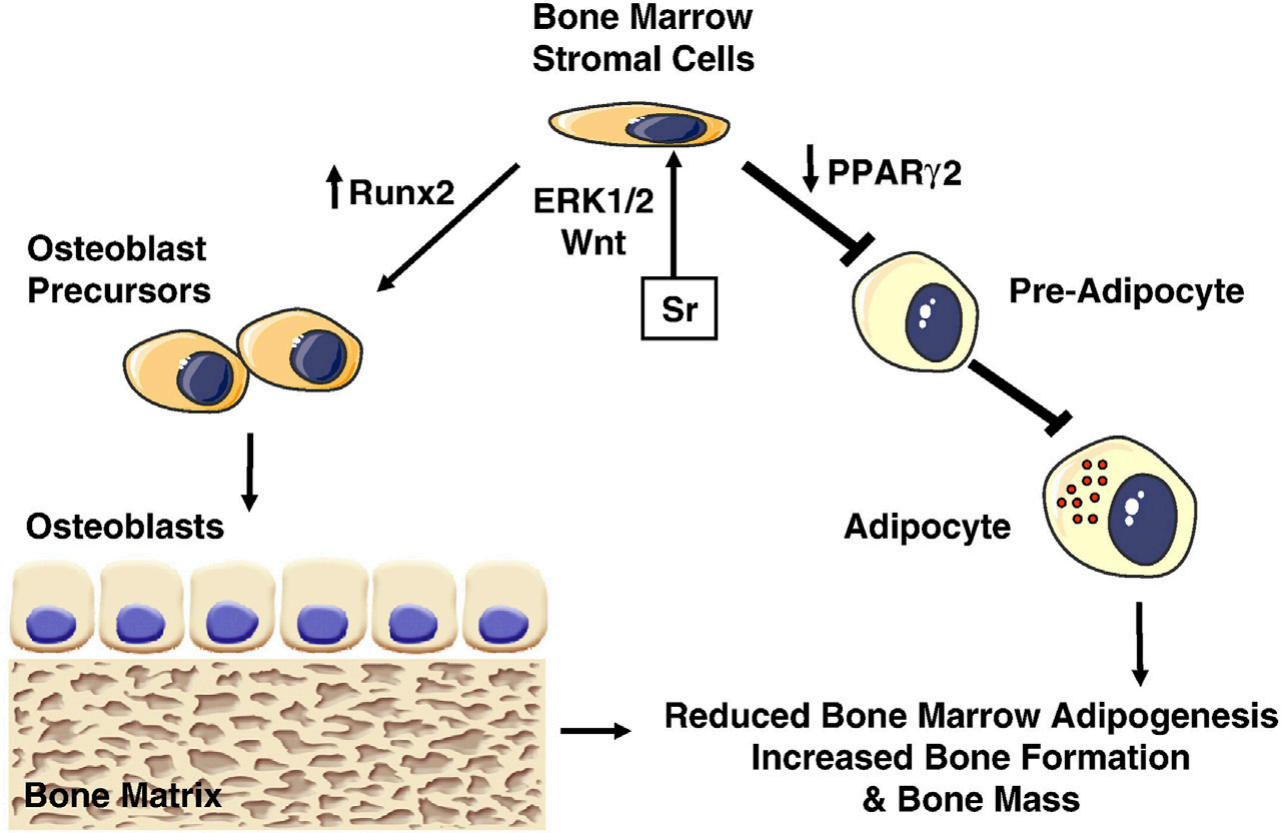
Effects of Strontium (Sr) on bone marrow stromal cells. Sr promotes osteogenesis by activating Wnt and ERK1/2-MAPK signaling pathways, which increase Runx2 and decrease PPARγ2 expression, inhibiting adipogenesis and increasing osteoblastogenesis (Saidak and Marie, 2012).

Mesenchymal stem cells (MSCs) are capable of multidirectional differentiation, which can differentiate into adipocytes, chondrocytes and a series of osteogenic-associated cells. The osteogenic differentiation of MSCs requires a complex process that includes osteoprogenitor cells, preosteoblasts, osteoblasts, and osteocytes during inducing osteogenic differentiation, which involves multiple types of intercellular and intracellular signaling transduction, such as signaling pathways, transcription factors, growth factors. Peroxisome proliferator-activated receptor-γ2 (PPARγ2) and Runt-related transcription factor (Runx-2) are essential genes regulating lipogenic and osteogenic differentiation of bone marrow mesenchymal stem cells (BMSCs). Increasing PPARγ2 expression and decreasing Runx2 expression promote lipogenic differentiation of BMSCs. While decreasing PPARγ2 expression and increasing Runx2 expression promote osteogenic differentiation of BMSCs (Akune et al., 2004) (Figure 1). A research compared Sr-SLA implant with SLA implant in regulating osteogenic transcription factors (β-catenin and osterix) and transcription factors critical for adipogenesis [CCAAT/enhancer-binding protein alpha (CEBPα) and PPARγ). And found that Sr-SLA implant significantly upregulated the expression of biochemical markers involved in the repair and regeneration of bone, such as β-catenin, osterix, alkaline phosphatase (ALP), osteomorphin and osteocalcin, while downregulated the expression of adipogenesis-related transcription factors CEBPα and PPARγ. Therefore Sr-SLA implant can promote osteogenic differentiation and inhibit adipogenesis of MSCs at the bone-implant interface compared to SLA implant (Choi and Park, 2018). Some researchers have reported that Sr^2+^ may reduce the expression of adipocyte genes (PPARγ2, CEBPα) and promote the expression of osteogenic genes [Runx2, ALP, osteocalcin (OCN) and bone sialoprotein (BSP)] by activating extracellular signal-regulated kinases (ERK)-MAPK and Wnt signaling to control the lineage allocation of MSCs, an effect that results in increased bone formation, decreased adipogenesis (Zhang et al., 2018b; Wang et al., 2019).

### 2.2 Effects of Sr on Osteoblast Cells

Sr^2+^ has been shown to have dual effects on bone metabolism, promoting bone formation while inhibiting the activity of bone resorption by osteoclasts (Pilmane et al., 2017). Sr^2+^ promotes the differentiation and proliferation of preosteoblasts and stimulates the secretion of new bone matrix by osteoblasts. Sr^2+^ prolongs the survival of osteoblast by inhibiting apoptosis through the ATK pathway and promotes early adhesion, proliferation, differentiation and matrix mineralization of osteoblast via integrin α2, integrin β1, adherents spot kinase, and ERK signaling pathways. In particular, osteocytes, as osteoclasts embedded in the mineralized matrix, produce sclerostin by expressing the SOST gene to inhibit osteogenesis. Strontium acts on osteocytes to reduce sclerostin thereby increasing bone mass. Xie et al. found that the dual behavior of Sr^2+^ on bone regeneration was related to the concentration of Ca^2+^: Sr^2+^ inhibited bone regeneration at low doses of Ca^2+^ and promoted bone regeneration at high doses of Ca^2+^ (Xie et al., 2018). Recently Vitro experiments have further demonstrated that strontium ranelate can promote osteogenic differentiation of adipose-derived stem cells by activating the calcium-sensing receptor (CaSR) on the surface of osteoblasts. Meanwhile, Sr^2+^ can promote osteoblastogenesis, matrix mineralization and calcium nodule formation by upregulating the expression of osteogenic genes such as ALP, OCN and BSP via activating mitogen-activated protein kinase (MAPK) signaling and ERK1/2 phosphorylation. However, the effect of Sr^2+^ to promote osteoblast proliferation and differentiation is dose-dependent. When the concentration of Sr^2+^ exceeded the optimal concentration range, its ability to promote osteoblast formation would be limited, and when it exceeded a certain threshold, there would be a toxic inhibition (Almeida et al., 2016; Liu et al., 2016). Almeida et al. have demonstrated that Sr^2+^ at a concentration of 1–10 mmol can effectively enhance the proliferation and survival activity of preosteoblasts, then accelerate the maturation of preosteoblasts into osteoblasts (Almeida et al., 2016).

### 2.3 Effects of Sr on Osteoclast Cells

Bone resorption is caused by an imbalance in skeletal homeostasis which starts with immature osteoclast precursors differentiate into osteoclasts under the action of specific cytokines. At the same time, the osteoclasts polarize and form a “ruffled membrane,” which forms an isolated extracellular microenvironment between the ruffled membrane and the bone surface called the “sealing zone.” Under the mediation of the vacuolar adenosine triphosphatase on the surface of the osteoclasts, osteoclasts begin to secrete resorptive organelle transporting acidifying vesicles and release HCl to the bone surface, causing an acidic environment. This acidic environment mobilizes the bone mineralized matrix leading to bone demineralization. Then the organic material freed from the bone surface is degraded by a lysosomal protease and cathepsin K (Cath-K), and the degradation products are endocytosed by osteoclasts and transported to the antiresorptive surface for release. Sr^2+^ released from biomaterials can alter the actin cytoskeleton of osteoclasts at the sealing zone, which reduces the bone resorption activity of osteoclasts by disrupting ruffled border formation and reducing the surface area available for proton exchange (Bakker et al., 2013). A recent study by Lourenço et al. found that Sr^2+^ not only reduced osteoclast adhesion and fusion with decreased functionality but also reduced expression of osteoclastogenesis-related genes such as tartrate-resistant acid phosphatase (TRAP) and matrix metalloproteinase-9 (MMP-9) to reduced bone resorption activity (Schumacher et al., 2016; Lourenço et al., 2019). Previous studies have shown that osteoprotegerin (OPG) can inhibit the binding of the receptor activator of nuclear factor κ-B (RANK) to the receptor activator of nuclear factor κ-B ligand (RANKL) on the surface of precursor osteoblasts by competitively binding to RANKL, thereby impairing the mature differentiation of precursor osteoblasts to osteoclasts. Sr^2+^ can increase OPG mRNA expression and downregulates RANKL expression of osteoblast that would block RANKL-induced activation of the nuclear factor kappa B (NF-κB) signaling pathway and impair osteoclastogenesis ultimately (Zhu et al., 2016). At the same time, OPG secreted by osteoblasts promotes apoptosis of osteoclasts through competitive binding with RANKL. Whereas it has been demonstrated Sr^2+^ also can inhibit the differentiation of pro-osteoclasts and promote the apoptosis of osteoclasts by activating protein kinase C-BII to reduce the bone resorption activity of osteoclasts in a dose-dependent manner (Bonnelye et al., 2008).

## 3 Molecular Mechanisms of Strontium

### 3.1 The Structure and Physiological Function of CaSR

The calcium-sensing receptor (CaSR) is a member of the G protein-coupled receptor superfamily that plays a crucial role in regulating Ca^2+^ concentration in extracellular fluid and maintaining skeletal homeostasis. CaSR is found not only in many tissues that regulate extracellular calcium concentration, such as parathyroid glands, kidneys and various bone cells but also in many tissues that are not directly involved in the regulation of extracellular calcium concentration, such as cardiovascular cells and mesenchymal stem cells (Pipino et al., 2014; Riccardi and Valenti, 2016; Marx et al., 2020). CaSR senses the Ca^2+^ concentration in the blood and stabilizes the Ca^2+^ level by regulating parathyroid hormone (PTH) secretion in the parathyroid glands and the reabsorption of Ca^2+^ in the kidneys and bones. CaSR can be present in different bone cells, such as osteoblasts and osteoclasts, and dominate the proliferation, differentiation and apoptosis of these cells (Marx et al., 2020). CaSR, has four metal-binding sites, one of which is populated with Ca^2+^ in both the inactive and active states of the receptor, while the other three sites are occupied with homologous Ca^2+^ only in the activated state (Ward et al., 1998; Cheshmedzhieva et al., 2021). These three sites are all capable of being bound by some specific divalent cations to activate CaSR, despite having different numbers and types of protein ligands, overall structures and charges sites (Geng et al., 2016; Cheshmedzhieva et al., 2021). Sr^2+^, with a similar structure to Ca^2+^, has the same valence state and significant affinity for more minor polar oxygen-containing ligands. Thus Sr^2+^ can fully activate the CaSr with similar effects to Ca^2+^ and modulate the downstream signaling pathways to promote osteoblastogenesis and inhibit the bone resorption activity of osteoclasts.

Skeletal homeostasis is maintained by coordinated activity between osteoblasts and osteoclasts, and CaSR is expressed on the surface of both osteoblasts and osteoclasts, suggesting the potential of specific divalent ions in regulating skeletal homeostasis (Ward and Riccardi, 2012). In terms of osteogenesis, Sr^2+^ activates CaSR on the surface of MSCs, inducing NFATc nuclear translocation to activate the Wnt signaling pathway, which upregulates Runx2, cyclooxygenase-2 (Cox-2) and ALP expression to promote osteogenic differentiation of MSCs. Sr^2+^ also can activate CaSR on the surface of osteoblasts, which would activate the downstream phosphatidylinositol 3-kinase (PI3K)/AKT signaling pathway and phosphorylating ERK1/2, promoting the nuclear translocation and transcription of β-catenin and glycogen synthase kinase-3β (GSK3β) to upregulate the expression of osteogenic-related genes ultimately (Rybchyn et al., 2011). In bone resorption, Sr^2+^ plays an integral role in the induction of osteoclast apoptosis through activation of the NF-κB signaling pathway. However, the mechanism of Sr^2+^ activated NF-κB signaling by CaSR is different from that of Ca^2+^ (Boyce et al., 2015). When stimulated by extracellular calcium, the CaSR activates phospholipase (PLC), which is responsible for the translocation of NF-κB from the cytoplasm to the nucleus in mature osteoclasts in an IP3-dependent manner. When stimulated by extracellular Sr^2+^, the CaSR also activates PLC, which stimulates activation of the diacylglycerol (DAG)-PKCβII signaling pathway in turn, promoting translocation of NF-κB from the cytoplasm to the nucleus in mature osteoclasts in an IP3-independent manner (Hurtel-Lemaire et al., 2009; Boyce et al., 2015). Sr^2+^ induced activation of NF-κB most likely in conjunction with other transcription factors, could intensify their respective effects, leading to enhanced apoptosis of mature osteoclasts.

### 3.2 Strontium-Related Signaling Pathway

#### 3.2.1 RANK/RANKL/OPG Signaling Pathway

Many studies have shown that Sr^2+^, a trace element that is highly relevant to the regulation of bone metabolism, has a “dual regulatory” effect, stimulating osteoblasts to secrete new bone matrix while inhibiting osteoclast activity to reduce bone resorption. The RANKL/RANK/OPG signaling pathway plays a crucial role in differentiation, activation and apoptosis of osteoclast (Uehara et al., 2020). RANK is highly expressed on the surface of both precursor osteoblasts and mature osteoclasts, while RANKL and OPG can be expressed in precursor osteoblasts and osteoblasts. Tumor necrosis factor (TNF) receptor-associated factor 6 (TRAF6) will be activated and recruited to bind with specific sites in the cytoplasmic when RANKL binds to RANK on osteoclast precursors. The formation of RANKL/RANK/TRAF6 complexes activates different intracellular cascade responses, including a series of signaling to initiate downstream JNK, NF-kB, ERK1/2, AKT which will activate downstream transcription factors such as AP1, NFATc1, thereby allowing the osteoclast precursors to differentiate into mature osteoclasts and break down the mineralized bone tissue (Park et al., 2017). On the contrary, OPG, a soluble decoy receptor, is a secreted glycoprotein secreted by osteoblasts and stem cells, whose structure is similar to RANK but does not contain a transmembrane structural domain (Lin et al., 2013). OPG can act as a negative regulator of osteoclasts by competitively binding to RANKL to block the RANKL/RANK signaling pathway, to inhibit osteoclast differentiation and maturation. Sr^2+^ binds to CaSr on the cell surface to upregulate the mRNA expression of OPG in osteoblasts y and MSCs firstly while downregulating the mRNA expression of RANKL. It increases the binding between OPG and RANKL and competitively inhibits the binding of RANK to RANKL in osteoclastic precursors, which in turn inhibits the maturation and differentiation of osteoclasts to reduce bone resorption (Reginster et al., 2014). Zhu et al. showed that Sr^2+^ could reduce the expression of RANK in macrophages, leading to downregulating the expression of Cath-K, MMP-9 and c-fos, which are related to the differentiation of macrophages to osteoclasts, thus reducing the number of osteoclasts and the resorption of bone tissue (Zhu et al., 2016).

The nuclear factor-kappa ligand (RANKL) receptor agonist will activate the NF-κB signaling pathway that would promote differentiation of MSCs to mature osteoblasts. The classical NF-KB signaling pathway involves activation of the IκB kinase (IKK) complex, which could phosphorylate IκB-α, resulting in activation of the NF-kB signaling pathway. NF-κB can form a complex with IκB-α when it is in an inactive state. However, IκB-α will be phosphorylated and degraded via the ubiquitination pathway when there are active molecules that stimulate NF-κB activation in the environment. They are transferred to the nucleus and initiate transcription of the target gene after depolymerization of the NF-κB and IκB-α complexes. A recent study showed that Sr^2+^ inhibited RANKL-induced activation of the NF-kB signaling pathway by inhibiting IkB-a phosphorylation and blocked nuclear translocation of NF-kBp65, leading to the inhibition of osteoclastogenesis (Mi et al., 2017; Huang et al., 2020). Zhang et al. prepared the Sr^2+^-doped submicrometer bioactive glass (Sr−SBG), an osteoimmunomodulatory bone repair material which showed that macrophages enhance the suppressive effect of Sr^2+^ on osteoclastogenesis and the inhibitory effect of Sr^2+^ may be attributed to the downregulation of TNFα and suppression of NF-κB pathway, resulting in the reduced recruitment and differentiation of osteoclast precursors (Zhang et al., 2016a).

#### 3.2.2 Wnt Signaling Pathway

The Wnt signaling pathway plays a critical role in all aspects of embryonic development, regulating key events in bone formation. Traditionally, Wnt signaling has been classified as canonical or non-canonical signaling. Canonical, β-catenin-dependent signaling results in activation of T-cell factor/lymphoid enhancer factor (TCF/LEF), whereas non-canonical signaling is β-catenin-independent which could be further divided into Wnt-Ca^2+^ pathway that leads to release of calcium as a second messenger and planar cell polarity pathway that leads to cytoskeletal rearrangement. β-catenin is that the key dominant of the canonical Wnt pathway. Once the Wnt/β-catenin signaling pathway is activated, Wnt protein binds to the frizzled protein (FZD) and low-density lipoprotein receptor-related protein 5/6 (LRP5/6) on the cell membrane surface, which control the formation of the β-catenin degradation complex by Axin axis protein recruitment of CKI-α and GSK-3β. Subsequently, the Dsh protein is phosphorylated to inhibit glycogen synthase GSK-3β, preventing the phosphorylation of β-catenin. The inhibition of GSK-3β induces β-catenin detachment from adenomatosis polyposis coli (APC), Axin, and GSK-3β (Nusse and Clevers, 2017). Then, unphosphorylated β-catenin accumulates in the cytoplasm and transfers to the nucleus, where it binds to the TCF/LEF transcription factor, at this point CBP/P300 is recruited to promote the expression of downstream osteogenic-related genes (Clevers and Nusse, 2012; Nusse and Clevers, 2017).

In the canonical Wnt pathway, Sr^2+^ activates calcineurin in osteoblasts leading to the nuclear translocation of NFATc1 and upregulating the expression of Wnt3a, which promotes the nuclear translocation of β-catenin and upregulates the expression of Runx2, ALP and another osteogenic gene to induce the proliferation and differentiation of osteoblasts (Yu et al., 2021). Meanwhile, strontium further enhances the nuclear translocation of β-catenin and promotes osteogenic factor expression by decreasing the expression of the sclerostin which is an inhibitor of the canonical Wnt pathway. In the non-canonical Wnt pathway, the activation of the NFATc1 can further promote the expression of Wnt5a and activate the downstream Ryk/RhoA, which can promote osteoblast differentiation and proliferation (Fromigué et al., 2010).

#### 3.2.3 Ras/MAPK Signaling Pathway

Mitogen-activated protein kinases (MAPKs) is a class of evolutionarily highly conserved serine/threonine protein kinases, containing more than a dozen proteins and belonging to three major families, namely the p38 MAPKs, the ERKs, and c-Jun amino-terminal kinases (JNKS). It has been reported that Sr^2+^ could activate MAPK signaling pathways in diverse cells, such as BMSCs and osteoblasts (Aimaiti et al., 2020). Sr^2+^ activates the osteogenic marker Ras, which activates mitogen-activated protein kinase kinase (MEK) and MAPK by phosphorylation in turn, to regulate various cellular physiological processes by activating transcription factors and protein kinases. Sr^2+^ enhances the activation of MAPK by mitogen in MSCs and promotes osteogenic differentiation of MSCs by increasing the phosphorylation of ERK1/2 and p38 to activate the expression of the downstream transcription factor Runx2 (Peng et al., 2009). Okita et al. demonstrated that Sr^2+^ could activate the ERK1/2 pathway through CaSR, leading to upregulating mRNA expression of collagen type 2 alpha 1 of chondrogenic differentiation gene to promote chondrogenic differentiation of dedifferentiated adipocytes (Okita et al., 2015). A recent study showed that Sr^2+^ promotes osteoblast differentiation by regulating the expression of the histone methylase Setd2 in activating the MAPK signaling pathway, and that is further demonstrated that Setd2 also regulates ERK activation to establish a positive feedback system during osteoblast differentiation, providing an alternative pathway for strontium to act in osteoblasts (Jia et al., 2017).

#### 3.2.4 NFATc Signaling Pathway

**FIGURE 2:**
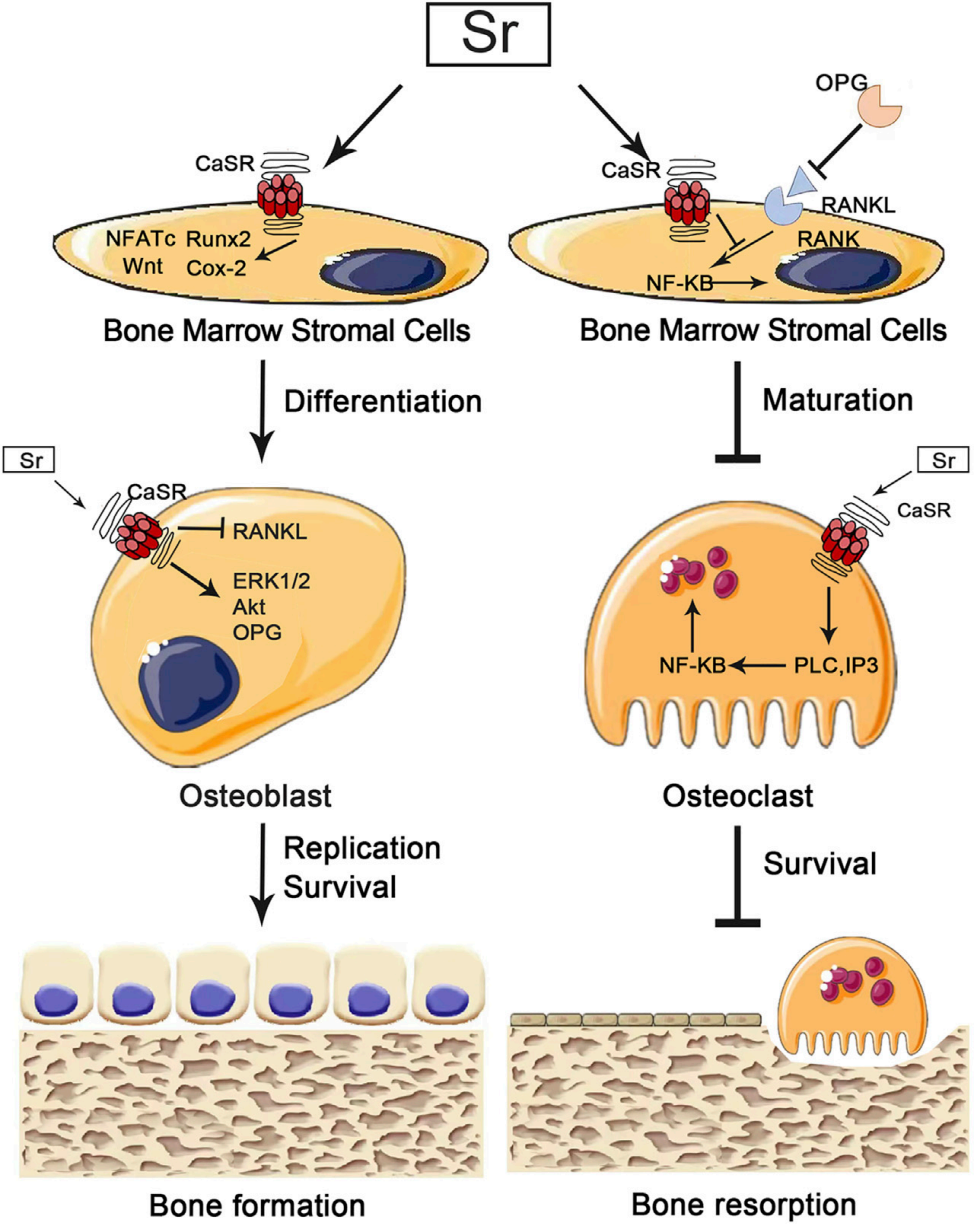
Implications for the pharmacological effects of calcium-sensing receptors (CaSr) in osteoblasts and osteoclasts. Strontium (Sr) promotes osteogenesis by activating CaSr to instruct osteoblasts in downstream pathways that promote osteoblast differentiation, replication and survival. Sr also inhibits bone resorption by activating CaSR and to instruct osteoclasts in downstream pathways that inhibit osteoclast maturation and survival.

The nuclear factor of activated T cells (NFATc), which plays a critical role in osteoblasts and osteoclasts, is expressed during osteoblast differentiation and bone formation to regulate the bone formation, osteogenic differentiation and remodeling (Kim and Kim, 2014). NFATc, usually found in the cytoplasm, is a highly phosphorylated transcription factor. The increasing level of intracellular calcium leads to activation of calcineurin which will dephosphorylate NFATc1 and translocates it to the nucleus, where NFATc1 binds to the promoter of the target gene to initiate gene transcription (Kumar and Roger, 2019). It has been shown that the expression of the Wnt gene can be promoted by activating NFATc1 in Sr-treated osteoblasts. Fromigue et al. showed that Sr^2+^ could activate calcineurin to encourage the increase of NFATc1 nuclear translocation and upregulate the expression of osteoblast phenotypic markers Runx2, ALP and ColI through NFATc1/Wnt signaling pathway to promote osteoblast proliferation, differentiation, and inhibit apoptosis (Fromigué et al., 2010). Simultaneously, NFATc1 promotes osteoclast differentiation and maturation by enhancing the expression of osteoclast-specific genes such as calcitonin receptor (CTR) and TRAP. Lee et al. found that when the Sr-nanocement was treated to pre-osteoclastic cells, the Sr-nanocement substantially downregulated *Nfatc1 in* mRNA level*, and* activity of TRAP, reducing the osteoclastogenesis and bone resorption capacity. In particular, the osteoclastic inhibition resulted in part from the interactive effect of osteoblasts which were activated by the Sr-nanocement through enhanced osteoprotegerin and the inactivated *Nfatc1 to* blockage of RANKL binding (Lee et al., 2021). The cellular CaSR-dependent mechanisms for Sr’s effect on osteoblast cells are summarized and shown in Figure 2.

#### 3.2.5 Other Signaling Pathways

The fibroblast growth factor receptor (FGFR) pathway is associated with skeletal development, which is observed in mice lacking FGF2 that abnormal bone trabeculae structure and reduced bone volume (Kao et al., 2020). The combination of FGF2 and FGFR promotes the proliferation of MSCs and upregulates the activity of ALP. FGF produced by osteoblasts is combined with FGFR on the surface of osteoblasts in an autocrine or paracrine manner to activate the downstream pathway, which promotes osteoblasts growth (Takei et al., 2015). Evidence shows that FGFR can react to Ca^2+^ and Sr^2+^ to regulate osteoblast growth through a mechanism independent of CaSR. Sr^2+^ can activate downstream signaling molecules of FGFR, including fibroblast receptor substrate 2 (FRS2) and ERK. The ability of FGFR-specific inhibitors to block the effect of Sr^2+^ on osteoblast proliferation suggests that the effect of Sr^2+^ on promoting osteogenesis is somewhat dependent on FGFR. In addition, the FGFR also promotes osteoblast proliferation in the presence of other cations such as Ca^2+^ and Al^3+^.

Smads is a signaling protein of the downstream signaling molecule of transforming growth factor (TGF)-β, which transmits TGF-β signals from extracellular to intracellular. TGF-β plays an essential role in maintaining the balance between bone formation and bone resorption, promoting osteogenic differentiation of BMSCs and inhibiting osteoclast formation. SMAD2/3 is triggered by TGF-β receptors and mediates TGF-β responses, while SMAD1/5/8 is triggered by the bone morphogenetic protein (BMP) receptors and transduces BMP signaling (Saito et al., 2018). BMP is a member of the TGF-β1 family, and the BMP2/Smad pathway is one of the extracellular signaling molecules that promote osteoblast differentiation and enhance bone formation. Zhi et al. showed that 5% and 10% Sr-CaS promote BMSC migration and osteogenic differentiation to improve the bone defects by modulating the TGF-β/Smad signaling pathway (Liu et al., 2019). However, Zhang et al. showed that Sr binds to BMP-2 to form the Sr-BMP2 complex rapidly that inhibits the Smad1/5/8 signaling pathway, resulting in suppression of BMP-2-induced ALP activity and downregulated expression of the ALP, Col I, OCN and Runx2 in protein level and mRNA level. These contradictory experimental results might be attributed to excessive strontium doping of the biomaterials, which leads to the release of large amounts of Sr^2+^ and reduces the biological activity of BMP-2 (Zhang et al., 2016b). Thus it is demonstrated the importance of Sr^2+^-doping concentration in biomaterials once again.

It has recently been shown that Sr^2+^ stimulates cartilage formation by activating the Hypoxia-inducible transcription factors-1α (HIF-1α) signaling pathway which would induce macrophage M2 phenotypic polarization and drive the formation of H-type vessels to promote bone reconstruction (Li S. et al., 2019; Cai et al., 2021). The H-type vessels have been shown to combine osteogenesis with angiogenesis by mediating the selective localization of Osterix (OSX) positive cells to perivascular sites and the subsequent differentiation of these osteoprogenitor cells. Some researches show that activation of the HIF-1α signaling pathway induces M2 phenotype polarization in macrophages (Park et al., 2020; Chen et al., 2021; Zhao et al., 2021). However, this seems to contradict the fact that the hypoxic environment can alter macrophage metabolic types and thus promote the polarization of macrophages toward the M1 phenotype (Van den Bossche et al., 2017; Mehla and Singh, 2019). Nevertheless, it is reasonable to speculate that there is a link between Sr^2+^, macrophage polarization and the HIF signaling pathway in bone reconstruction and vascular regeneration. Thereby we could explore this further in the future.

## 4 Immunological Mechanism of Strontium for Osteogenesis

### 4.1 Macrophages in Bone Immunity

#### 4.1.1 The Origin of Macrophages

There are two primary sources of macrophages: the circulating monocyte-derived macrophages in the peripheral blood and the tissue-resident macrophages (TRM). The former developed from monocytes of the bone marrow and colonized the bone marrow to produce bone marrow monocytes that seed the blood continuously throughout life, whereas the latter derived from the embryonic yolk sac and fetal liver precursors (Watanabe et al., 2019). Embryonic-derived macrophages are transferred to different parts of the body during subsequent growth and proliferate into TRM (Gordon and Plüddemann, 2017). TRM are variable in various tissues, such as alveolar macrophages in the lung and Kupffer cells in the liver. Bone-resident macrophages are divided into erythroblastic island macrophages, hematopoietic stem cell niche macrophages and skeletal macrophages (sMΦ). sMΦ, also called osteal macrophages or osteomacs, have been reported to contribute to bone homeostasis and regeneration significantly (Batoon et al., 2019; Wan Z. et al., 2020). Furthermore, bone marrow resident macrophages which have a predominantly embryonic ontogeny and a high capacity for self-renewal do not require constantly repopulated from monocyte-derived macrophages in the blood stream. Bone marrow provides a large number of monocytes during inflammation, with a portion of monocytes migrating through the blood circulation to various tissues and gradually differentiating into macrophages during the migration process (Horwood, 2016). This source of macrophages constantly replenishes embryonic-derived macrophages (De Schepper et al., 2018; Shaw et al., 2018). Thus, at any given point in time, the macrophage population of any tissue is composed of different proportions of embryonic-derived and bone marrow-derived macrophages.

#### 4.1.2 Macrophage Polarization

Macrophage polarization is mainly related to three aspects: 1) Exogenous factors (cytokines): When exposed to a microenvironment containing unique cytokines causes macrophage polarization. For example, when a TH1-driven immune reaction occurs *in vivo*, CD4^+^ T cells secrete IFN-γ to induce M1 macrophages. TH2-driven immune responses in which interleukin-4 (IL-4) and IL-13 are produced induce M2 macrophages (Rőszer, 2015; Junttila, 2018). Therefore, we usually expose macrophages to the corresponding cytokine environment to induce polarization *in vitro* experiments. 2) Non-cytokine exogenous factors: macrophages that are the first line of defense in the innate immune response have to respond rapidly to pathogenic foreign substances or tissue damage signals to initiate the inflammatory cascade response. Most of the M1 macrophages are directly related to the resistance towards infection, as these cells require rapid bactericidal activity and most of the injured tissue is in a hypoxic microenvironment. *In vitro* studies have shown that M1 macrophages display increased anaerobic glycolysis and pentose phosphate pathways to ensure rapid energy production (Van den Bossche et al., 2017). In contrast, M2 macrophages are associated with tissue remodeling, repair, and wound healing that requires a continuous intracellular energy supply. Therefore, the metabolism of M2 macrophages is more oriented towards oxidative phosphorylation and fatty acid oxidation during mitochondrial respiration (Viola et al., 2019). The effect of primary metabolic pathways on macrophage polarization is only coming to the fore. More studies are needed to show their relationship with macrophage polarization before it can be determined. 3) intrinsic pathway of macrophage development and the tissue environment: Whether the developmental origin of macrophages affects the final polarization endpoint has not yet been determined. However, some studies suggest that macrophage polarization is a conserved process, independent of the origin of the macrophage (van de Laar et al., 2016). We can determine that most tissue macrophages are replaced by macrophages differentiated from monocytes in the circulatory system during tissue transplantation. The rapid depletion of tissue-resident macrophages after the onset of infection is replenished by bone marrow-derived macrophages. However, this massive turnover of macrophages still preserves the normal function of the tissues. Therefore, if the activation of macrophages and thus function is more oriented towards the influence of the tissue environment, from this point, the developmental origin of macrophages is less important for the final polarization phenotype (Murray, 2017).

**FIGURE 3:**
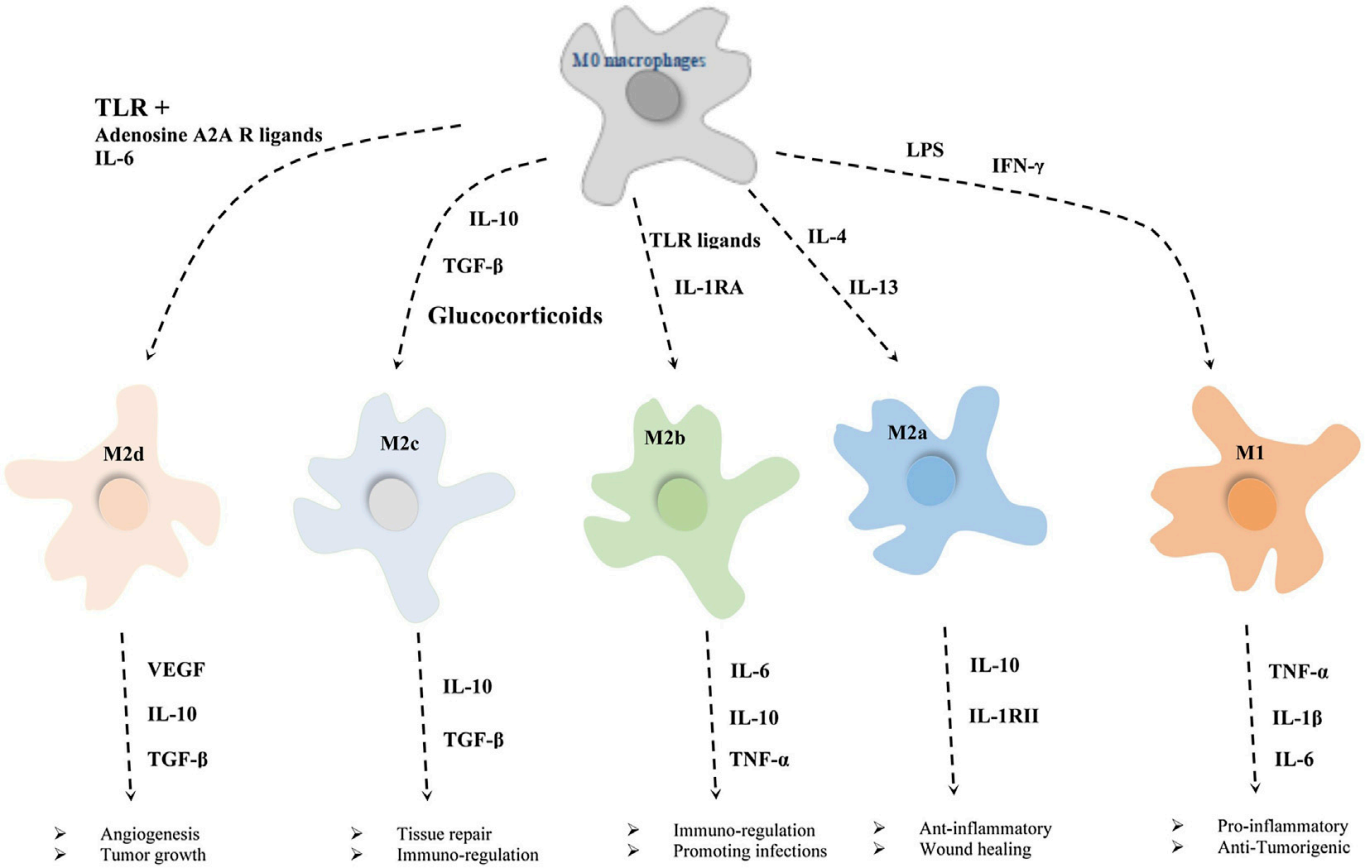
Different exogenous factors induce the direction of macrophage polarization and the roles of different macrophages in various processes (Ping et al., 2021).

The dichotomous system classifies macrophages into M1 macrophages activated by lipopolysaccharides (LPS) stimulation and M2 macrophages activated by IL-4 and IL-13 stimulation (Xie et al., 2020). The M1 macrophage are classified as pro-inflammatory or classically activated, while the M2 macrophages were classified as an anti-inflammatory or alternatively activated (Spiller and Koh, 2017; Najafi et al., 2019). M1 macrophages, which are identified by the staining of the macrophage marker CD68 and CD80, mainly secrete pro-inflammatory cytokines such as Tumor necrosis factor-α (TNF-α) and IL-1, IL-6, which are responsible for the recruitment of immune cells at the trauma site, elimination of pathogens, and initiation of the acute inflammatory response (Jin et al., 2019; Guo et al., 2020b; Yang et al., 2020). Therefore, M1 macrophages lay the foundation for subsequent bone tissue repair; M2 macrophages, which are identified by the co-staining of CD68 and CD206, mainly secrete anti-inflammatory cytokines such as IL-4, IL-10 and transforming growth factor-β (TGF-β), which play an important role in promoting tissue repair as well as remodeling (Chen et al., 2014; Lee et al., 2019). However, there is no clear dividing line between the two types of macrophages: M1macrophages can also secrete some anti-inflammatory cytokines, but M2 macrophages make more. Meanwhile, it is wrong to cover the process of macrophage activation and polarization only by relying on M1 and M2 types. The process of macrophage polarization is far more complex than we thought, with no scientific basis to justify the dichotomous model of macrophage polarization based on *in vitro* stimulation modalities. A “spectrum” of macrophage polarization may exist involving macrophages with different metabolic, inflammatory profiles and roles in host defense against various pathogens, wound healing and inflammation regression (Mosser and Edwards, 2008). For example, according to the vitro stimulation experiments, the M2 phenotype can be further subdivided into M2a (produced by IL-4/IL-13 stimulation), M2b (produced by combined stimulation of immune complex and toll-like receptor/IL-1R agonist), M2c (produced by IL-10,TGF-β and glucocorticoids stimulation), M2d (TLR of IL-6 or adenosine A2A receptor ligands) (Mantovani et al., 2004; Horwood, 2016). Although all these subtypes show anti-inflammatory properties; however, M2a and M2b exhibit immunomodulatory effects, while M2c is related to immunosuppressive phenotype and extracellular matrix remodeling. M2d phenotype macrophages enhance the growth and angiogenesis of tumors (Figure 3) (Abdelaziz et al., 2020; Ping et al., 2021). However, there is still great controversy about macrophage polarization: Whether macrophage polarization is achieved by changing their phenotype or by recruiting a new group of phenotypic macrophages to reach the target area needs to be further investigated (Sica and Mantovani, 2012).

#### 4.1.3 Macrophages in the Process of Bone Reconstruction

**FIGURE 4:**
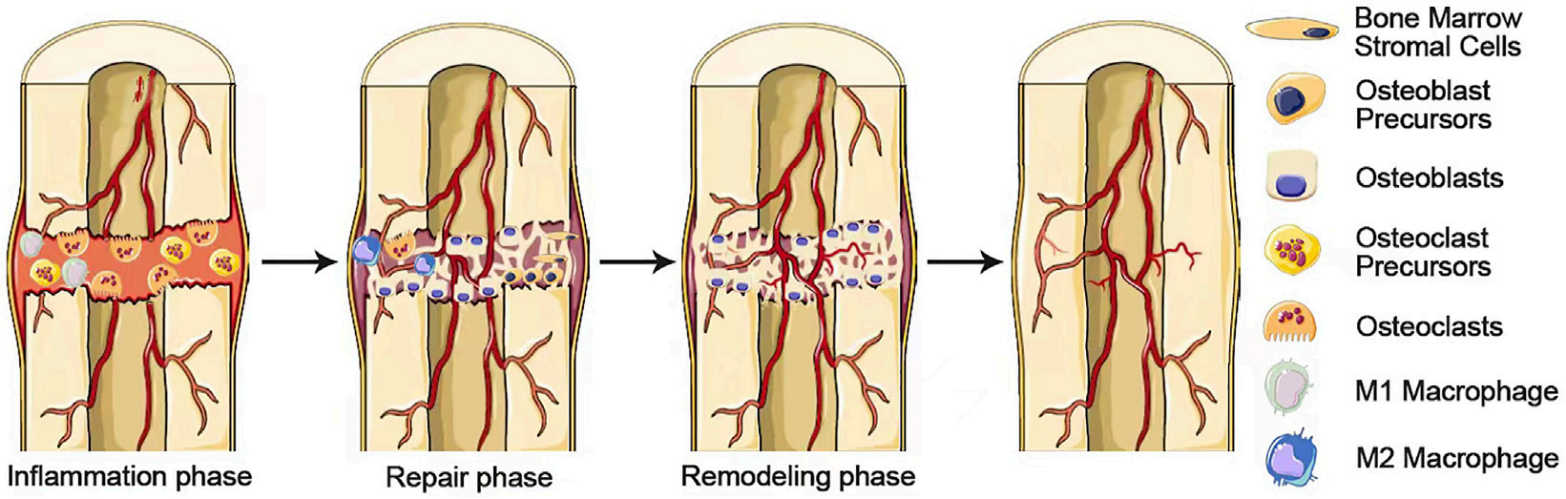
A complex and well-orchestrated bone repair process involving three phases. Inflammation, repair, and remodeling phases.

Bone reconstruction is a complex and ordered process consisting of three main sequential phases: inflammation, repair and remodeling. The communication mechanism by which bone formation follows bone resorption is described as coupling, in which osteoclasts drive remodeling as an engine and osteoblasts secrete the matrix subsequently as the cellular carriers. These processes occur simultaneously in multiple locations in the skeleton, with the sites of bone formation and bone resorption activity becoming basic multicellular units (BMUs) (Delaisse, 2014). The inflammatory phase begins with the partially differentiated osteoclast progenitors migrating to the bone surface, where osteoclast progenitors mature into multinucleated osteoclasts (Wittkowske et al., 2016). The multinucleated osteoclasts remove the old or damaged bone surface. The repair phase is initiated when monocytes appear on the bone surface. Monocytes emit signals to direct the recruitment and differentiation of osteoblasts and prepare the bone surface for osteoblasts to initiate the bone remodeling phase (Borciani et al., 2020). During the remodeling phase, osteoblasts secrete bone matrix on the bone surface until the site of bone resorption is entirely replaced by new bone (Figure 4). Upon completion of this phase, flattened lining cells cover the surface and an extended rest phase begins until a new reconstruction cycle begins. Each phase of the reconstruction cycle has different length: Inflammation may last for about 2 weeks, the repair stage may last for 4 or 5 weeks, while remodeling can last up to 4 months until the new structural units of skeleton are fully formed (Hadjidakis and Androulakis, 2006).

After implantation of the bone substitute material into the injured area, the change of blood cause tissue fluids, proteins and cells derived from the surrounding tissues to attach to the surface of the material Immediately, inducing the formation of a thrombus which is comprised of platelets, white and red blood cells as well as fibrin (Perić Kačarević et al., 2020). The neutrophils and monocytes that initiate the acute inflammatory response are the first to reach the damaged area when stimulated by biomaterial implantation. Monocytes differentiate into macrophages as they enter the circulation. Macrophages, an essential regulator of innate and adaptive immunity, are critical cells recruited by polymorphonuclear leukocytes (PMN) upon detection of pathogens (Parisi et al., 2018). The role of macrophages is mainly through the following ways: 1) Eliminating cellular debris, dead cells and pathogens by phagocytosis; 2) initiating appropriate or pathogenic inflammatory responses (mainly by M1 macrophages); 3) secreting proteins that stimulate wound healing, such as cytokines and enzymes (specifically by M2 macrophages) (Naruphontjirakul et al., 2021). Increasing evidence suggests that macrophages associated with implanted biomaterials play an important role in bone formation, as macrophages take a dynamic role in initiating the recruitment of MSCs and vascular progenitor cells engaged in angiogenesis (Nair and Tang, 2017; Niu et al., 2017; Yang et al., 2018; Xie et al., 2020; Niu et al., 2021).

M1 macrophages recruit MSCs, vascular progenitor and osteoprogenitor cells to the fracture site by secreting stromal cell-derived factor 1 (SDF1), CCL2 and CXCL8 in the early stage of inflammation (Wang et al., 2018). It has been shown that depletion of M1 macrophages results in a significant decrease in pro-inflammatory cytokines such as IL-6, TNF-α and interferon gamma-induced protein (IP-10), leading to impaired fracture healing (Hozain and Cottrell, 2020). Inflammation seems to act as a switch to turn on the healing phase is indispensable (Spriano et al., 2018). Thus the presence of M1 macrophages during the initial inflammatory phase is essential for normal bone healing. Nonetheless, there must be a strictly time-limited phase of inflammation. Otherwise, the injury with excessive acute inflammation and the pathological condition of chronic systemic inflammation can negatively affect fracture healing and lead to delayed healing (Spriano et al., 2018). For example, a research using lipopolysaccharide to induce acute systemic inflammation in mice showed a reduction in the quantity and quality of regenerated femurs (Behrends et al., 2017). M1 macrophages that could not switch to the M2 phenotype in time would secrete pro-inflammatory cytokines continuously, resulting in delayed healing and chronic inflammation (O'Brien et al., 2019). Nevertheless, premature M2 macrophages may produce excess fibrotic cytokines that form a fibrous capsule on the surface of the biomaterial, thereby adversely affecting implant integration (Wynn and Vannella, 2016; Zheng et al., 2018). Consequently, ending the inflammatory phase at the right time is necessary to form a favorable environment for bone regeneration. Despite there is no consensus on which macrophage phenotype is most favorable for osteogenesis, there is no doubt that the induction of an appropriate immune environment at a specific time by biomaterial is essential for bone healing. M1/M2 macrophages play an important role at different stages of the bone healing process and promoting a specific macrophage phenotype properly at an optimal time is better for bone regeneration than promoting a single phenotype directly or promoting both phenotypes simultaneously (Chen E et al., 2018). Kim et al. showed that promoting the polarization of M1 macrophages (but not the M2 macrophages) on the first day of biomaterial implantation and the timely conversion of M1 macrophages to the M2 phenotype on the subsequent third day was necessary to enhance the regenerative effect (Kim and Tabata, 2016, 2017; Liu et al., 2021). Similarly, Nathan et al. used LPS-induced M1 macrophages co-cultured with MSCs and then added IL-4 for various durations to induce the M2 phenotype. The results showed that the pro-inflammatory environment in the presence of M1 macrophages for 72–96 h was critical for stromal mineralization. Interestingly, the optimal timing of the M1 to M2 transition in MSC osteogenesis is sex-dependent: the optimal transition time is 72 h for females versus 96 h for males. This sex-related difference in MSC osteogenesis may be caused by differential expression levels of steroid receptors that mediate stem cell proliferation and differentiation (Nathan et al., 2019). Therefore, attempts to direct macrophages to promote bone reconstruction must strictly control macrophage phenotype switching sequence and time to coordinate the transition between the inflammatory and repair phases. The degree of polarization should also be strictly controlled, as excessive induction of M1 or M2 phenotype can have a negative impact on the final bone healing (Spriano et al., 2018). Most of the existing studies focus on whether strontium can induce macrophage polarization to obtain a better effect on bone healing, whereas the influence of the time and degree of macrophage polarization on the bone healing has been neglected. Here we summarize the existing studies on the time of polarization in bone healing and conclude that the optimal polarization time for macrophage conversion from M1 to M2 phenotype should be day 3–4 after implantation of the biomaterial. The future development of strontium-doped biomaterials should focus on how to combine the immunomodulatory function of strontium with the time factor to achieve precise modulation.

### 4.2 Strontium Induces Macrophage Polarization for Osteogenesis and Angiogenesis

The regulation of immune cells can influence bone remodeling and regeneration (Schlundt et al., 2015). Before angiogenesis and osteogenesis, the early inflammatory response of immune cells to the biomaterial surface determines the fate of the implant in the body. The acute inflammation occurring at the implantation site during the early stages of bone regeneration can recruit immune cells such as stem cells, T cells and monocytes to reach the injury site, which could release cytokines and chemokines such as TNF and IL-6 to facilitate the differentiation of MSCs into osteoblasts (Gibon et al., 2016). However, chronic inflammation that produces excessive pro-inflammatory cytokines may suppress osteoblastogenesis and bone formation. Therefore, the use of biomaterials to terminate inflammation and create a favorable local immune microenvironment at the right time will facilitate subsequent osteogenesis. Macrophages, as innate immune cells that reach the site of inflammation early, promote osteogenesis through regulation of macrophages is mainly manifested in two ways: on the one hand, macrophages are used to remove pathogens during the inflammatory phase and exclude unfavorable disturbances in the osteogenesis process. On the other hand, the cytokines secreted by macrophages can be used to form a favorable osteogenic microenvironment.

Numerous studies have shown that strontium can promote bone reconstruction by regulating macrophages (Table 1). Sr^2+^ induces the conversion of macrophages to the M2 phenotype to upregulate the expression of growth factors associated with tissue regeneration, such as vascular endothelial growth factor (VEGF), platelet-derived growth factor-BB (PDGF-BB), IL-10, TGF-β1, BMP-2, among which IL-10 promotes osteogenetic differentiation of osteogenesis-related cells by activating the p38/MAPK signaling pathway, and TGF-β1 and BMP2 intensely induce osteogenic differentiation of osteoblasts (induction of migration, recruitment and osteogenic differentiation of MSCs), creating a favorable immune microenvironment for bone tissue regeneration (Yuan et al., 2017; Chen J et al., 2018; Jin et al., 2019; Lee et al., 2019). M2 macrophages promote the expression of ALP, osteocalcin and osteopontin (maturation markers of osteoblasts) in MSC to enhance MSC osteogenic differentiation (Franz et al., 2011; Chen et al., 2012; Chen et al., 2014; Lee et al., 2019). At the same time, Sr^2+^ can inhibit macrophage differentiation to osteoclasts by down-regulating TRAP, MMP-9 and CKT expression (Wu T. et al., 2021). Xu et al. (2021) fabricated Sr^2+^ incorporated micro\nano rough titanium surface (Sr-SLA) and immunoreaction of macrophages was further investigated. The Vivo experience showed that compared to SLA implants, less classically activated M1 macrophages infiltration and more alternatively activated M2 macrophages infiltration were observed at the implantation site of Sr-SLA implants, accompanied with a more bone formation. The expression levels of ALP, BMP2, IL-10 and other osteogenesis-related factors were significantly higher in the Sr-SLA group than in the SLA group. Western blot results showed that Sr-SLA increased the phosphorylation of ERK, while there was no obvious difference in the phosphorylation of JNK and p38, which suggests that Sr-SLA induced polarization of macrophages may be associated with the activation of the ERK signaling pathway (He et al., 2021). Likewise, Richardson et al. (2015) showed that the activation of the ERK signaling pathway is necessary for the differentiation of monocyte-macrophage. If blocking the activation of the ERK signaling pathway during the early stages of macrophage differentiation will inhibit the polarization of macrophages to the M2 phenotype. The optimal amount of Sr^2+^ doping seems to be different for diverse biomaterials in terms of bone healing. For example, Zhang et al. (2018a) prepared bioactive glasses with strontium doping amounts of 0%, 5%, 10%, and 15%, and found that 10% Sr-MNBG had the best pro-macrophage M2 polarization and the best bone-producing effect of macrophage secretory components at this concentration. Li et al. (2012) reported that MSC proliferation was suppressed in the presence of Sr^2+^ concentrations between 0.1 and 1 mM when MSCs were cultured under basal conditions for 15 days, but Lourençoa et al. (2019) showed that MSC proliferation was promoted in the presence of Sr^2+^ concentrations between 0.5 and 1 mM when MSCs were cultured under basal conditions for 14 days. This may be caused by the different scaffold materials they use, so confirming the optimal Sr^2+^ concentration range of different biomaterials and utilizing Sr^2+^ to promote bone healing through macrophages will be one of the issues that must be broken through of strontium-doped bone repair materials (Aimaiti et al., 2017). Although the optimal strontium concentration to be introduced in biomaterials is still controversial. Numerous *in vitro* studies have shown that Sr concentrations above 1 mM stimulate osteogenesis, but it is best to consider the results of clinical trials. Combined with the available relevant studies recommended here for an effective range below 500 mM (Bonnelye et al., 2008; Peng et al., 2009; Guo et al., 2016).

**TABLE 1:** Summary of studies about strontium induces macrophage polarization for osteogenesis and angiogenesis.

Material	*In Vivo*/*In Vitro* evaluation	Key findings	References
Sodium titanate (ST) nanorods doped with different Sr content	*In vitro*: macrophages or MC3T3 were respectively seeded on the bare and nanorods-arrayed Ti discs	*In vitro*: Sr doped arrays accelerate phenotypic transformation of the adhered macrophages towards M2 phenotype	Yu et al. (2022)
	*In vivo*: implantation on Ø1.5 × 4 mm sized holes on both sides of femoral condyles in rat	*In vivo*: The Sr-doped nanorods arrays significantly enhance bone-implant contact in comparison with ST	
Strontium containing sol-gel derived BGNPs (Sr-BGNPs)	*In vitro*: RAW264.7 cells were seeded on conditioned medium containing the NPs at concentration range from 0 to 250 μg/ml	*In vitro*: macrophages polarised towards the M2 population in the presence of Sr-BGNPs rather than the pro-inflammatory M1 population	Naruphontjirakul et al. (2021)
Rg1/SrP/SG-based organic–inorganic biocomposite scaffolds	*In vitro*: HUVECs and M1 macrophages were cultured in extracts from Rg1/SrP/SG with different strontium-doped content	*In vitro*: the expression levels of VEGF and bFGF genes of HUVECs were upregulate; the protein levels of MMP9, CTK and TRAP of RAW264.7 were inhibited	Wu et al. (2021a)
	*In vivo*: implantation on rat with critical-sized calvarial defects	*In vivo*: most areas of the defects were filled with the newly generated bone at 12 weeks	
strontium incorporated micro/nano rough titanium surfaces (Sr-SLA)	*In vitro*: RAW264.7 cells were seeded on Ti surfaces	*In vitro*: more M2 surface marker CD163 expression on Sr-SLA	Xu et al. (2021)
	*In vivo*: Sr-SLA implantation on male Sprague-Dawley rats bilateral tibiae defect	*In vivo*: more copious collagen deposition was observed around the implants and bone island formation in the thread region at 7 days after implant	
a newly sustained release system consisting of Sr ion-loaded sodium titanate nanorods (STSr)	*In vitro*: biological evaluation with HUVECs and Raw 264.7 cells	*In vitro*: STSr significantly promoted the angiogenesis and formation of CD31^hi^Emcn^hi^ vessels by modulating the transformation of M1 macrophages toward M2 macrophages	Guo et al. (2020a)
	*In vivo*: implantation on rat femoral condyle implant model	*In vivo*: Accompanied with enhanced vascularization, improved bone formation and osseointegration were observed	
Porous scaffold made of Ti with Sr^2+^ and Ag^+^ (AH-Sr-AgNPs)	*In vitro*: biological evaluation with MC3T3 cells and Raw 264.7 cells	*In vitro*: Expression of *CD206* was significantly higher (M2 phenotype markers) and promotion of pre-osteoblast differentiation of MC3T3 cells with higher expression of ALP, RUNX2, and COL1	Li et al. (2019a)
	*In vivo*: implantation on infected New Zealand rabbit femoral metaphysis defect	*In vivo*: complete bone coverage and penetration into the pores of AH-Sr-AgNPs	
Sr-rich HAp microspheres and an RGD (arginine-glycine-aspartic acid)-modified alginate hydrogel	*In vitro*: MSCs were cultured in conditioned medium with different SrCl2 content	*In vitro*: a progressive increase in the number of MSCs and ALP activity	Lourenço et al. (2019)
	*In vivo*: male BALB/c mice with air-pouch model of inflammation	*In vivo*: a statistically significant increase (*p* < 0.05) in the percentage of F4/80/CD206 cells (M2 phenotype markers); A thin fibrous capsule with low infiltration of inflammatory cells within the scaffolds	
Strontium- substituted micro/nano bioactive glasses (Sr- MNBG) with 0, 5%, 10%, and 15% molar percent of strontium element	*In vitro*: macrophage were cultured in conditioned medium with Sr- MNBG	*In vitro*: 10% and 15% molar percent of strontium element could further promote macrophage polarization toward M2	Zhang et al. (2018a)
	*In vivo*: implantation on rat femoral condyle implant model	*In vivo*: more M2 macrophages around 10% Sr- MNBG after implantation in the body, and better bone repair effect was found in the 10% Sr- MNBG group	
Sr-substituted BG microsphere (SrBGM)	*In vitro*: HUVECs and Raw 264.7 cells were cultured in extracts from SrBGM with different strontium-doped content	*In vitro*: the RAW presented a trend towards to M2 phenotype; SrBGM regulated macrophage could significantly enhance the angiogenesis of HUVECs	Zhao et al. (2018)
	*In vivo*: implantation on rat Cranial defects	*In vivo*: All the scaffolds had closely attached to the skull surface without any space	
Commercially pure Ti disks with surface functionalized with Sr ions	*In vitro*: biological evaluation with mouse J774.A1 macrophages	*In vitro*: Induction of regenerative M2 macrophage phenotype of J774.A1 cells in nanostructured Ti surfaces	Lee et al. (2016)

Although Sr^2+^-doped biomaterials has been showed that could promote bone formation by inducing macrophage polarization to the M2 polarization which are more closely related to osteogenesis by secreting anti-inflammatory cytokines, M2 macrophages also secrete fibrogenic cytokines such as TNF-α, TGF-β1, TGF-β3, and PDGF-BB, which may lead to pathological fibrosis and delayed bone healing. Chen et al. injected different doses of IL-4 into a pre-implanted decellularized bone matrix to induce M2 macrophages in the environment, thereby improving bone formation (Wittkowske et al., 2016). However, it has been shown that lower doses (10 ng) of IL-4 led to better bone formation than higher doses (50 and 100 ng) of IL-4 (Chen et al., 2017). The reason may be that the lower dose could have produced an optimal M2/M1 macrophage ratio, whereas the higher dose resulted in excessive M2 macrophage that leads to the formation of fibrosis (Wynn and Vannella, 2016; Lourenço et al., 2019). Wang et al. (2017) found that BMSCs showed increased migration and osteoblastic differentiation in culturing BMSCs with M1 macrophage-conditioned media compared to the macrophage-free media, which confirmed that M1 macrophages also play a positive role in regulating osteogenesis. Lu et al. (2017) showed that M1 macrophages enhance osteogenesis via the COX-2 and prostaglandin-E2 (PGE2) pathway. In addition, some recent studies have found that M1 macrophages can also promote osteogenic differentiation of MSCs through the induction of transcription factors CEBPβ and CEBPδ by the oncostatin M (OSM) (Liu et al., 2020). OSM which has been identified as specific macrophage factors that promote the osteogenic potential of MSCs, may act in the early stages of intramembranous osteogenesis and is responsible for regulating recruitment, proliferation and sinking of the mineralized matrix of MSCs by activating STAT3 signaling. Thus it is the conversion pattern of macrophages, not a specific phenotype of cells, that determines the success of bone repair.

The degree of vascularization of the biomaterial implantation site is one of the critical factors in promoting bone regeneration. During bone reconstruction, the differentiation of MSCs to osteoblasts is accompanied by invasion of the capillaries network which can serve as a template for bone development. Efficient vascularization not only provides the necessary nutrients and oxygen to the damaged site but also is a prerequisite for the repair of bone defects by MSCs and preosteoblasts (Kargozar et al., 2020; Ding et al., 2021). In previous studies, Sr^2+^ has been reported to promote angiogenesis by stimulating osteoblasts to secrete angiogenesis-related cytokines (Zhao et al., 2015; Mao et al., 2017; Offermanns et al., 2018). However, it should be noted that an acute inflammatory response occurs at the site of injury immediately after implantation of biomaterials, while osteoblasts are recruited to the site by cytokines released by inflammatory cells subsequently. Thus osteoblasts have a limited role in early angiogenesis around implanted materials at the early stage (Schmidt-Bleek et al., 2015). However, macrophages, which accumulate at the site of damage as inflammatory cells early in inflammation, play an essential role in angiogenesis. After the fracture, the destruction of blood vessels creates a hypoxic environment, and hematoma formation isolates the injury site from perfusion, further increasing local hypoxia and decreasing pH (Walters et al., 2018). The hypoxic and acidic microenvironment attracts the aggregation of macrophages, which survive in the hypoxic environment by regulating their metabolism to produce adenosine triphosphate (ATP) in a non-oxygen-dependent manner and gradually polarized to the M2 phenotype (Murdoch et al., 2004; Ke et al., 2019; Wu et al., 2019). Stimulated by hypoxia-inducible factor HIF-1, macrophages would secrete VEGF, which stimulates the generation and chemotaxis of endothelial cell precursor cells. The healing-promoting role played by macrophages extends from the initial inflammatory phase through to the regenerative and remodeling phases of repair.

Sr^2+^ has been reported to have the function of promoting angiogenesis (Zarins et al., 2016). Sr^2+^ released from Biomaterials could promote the expression of pro-angiogenic factors such as VEGF, basic fibroblast growth factor (FGF) and MMP-2 (Wang et al., 2014). Zhao et al. prepared strontium-substituted submicrometer bioactive glass (Sr-BGM) and SBG without strontium-substituted to investigate the different effects of the induction of angiogenesis in HUVECs. The results showed that SrBGM has no significantly different effect on regulating the angiogenesis of HUVECs compared with BGM under a normal growth medium without RAW264.7 (macrophage cell). Furthermore, compared to BGM + RAW264.7 conditioned medium, SrBGM + RAW264.7 conditioned medium obviously strengthens the angiogenesis ability of HUVECs. Meanwhile, *in vivo* experiments confirmed that SrBGM could promote early vascularization by inducing the production of M2 macrophages at the implantation site (Zhao et al., 2018). This demonstrates that Sr^2+^ does not directly promote angiogenesis but could enhance early angiogenesis by mediating the polarization of macrophages and have wide application in bone regeneration (Guo et al., 2020a; Guo et al., 2020b). During tissue repair, M1 macrophages dominate in the early period (days 1–5) after injury, while M2 macrophages dominate in the later period (days 7–14) (Zhao et al., 2018; Pajarinen et al., 2019; Niu et al., 2021). Previous studies have suggested that M2 macrophages are considered as angiogenic phenotypes due to their recruitment of MSCs and vascular progenitor cells involved in angiogenesis during bone healing, while M1 macrophages have little effect on angiogenesis. Nevertheless, Spiller et al. (2015) showed that M1 and M2 macrophages both promote vascularization in different ways and are both necessary for angiogenesis. M1 macrophages initiate angiogenesis by secreting growth factors such as VEGF. M2 macrophages maintain the stability of vascular network formation by secreting related growth factors and coordinating the assembly of extracellular matrix. M2a macrophages can participate in the formation and stability of the vascular system and promote endothelial cell anastomosis by secreting PDGF-BB, and placenta growth factor (PIGF) at high levels; M2c macrophages can secrete MMP and FGF at high levels to participate in vascular remodeling (Jetten et al., 2014). Guo et al. fabricated Sr^2+^-loaded sodium titanate nanorods (STSr) and investigated their effects on angiogenesis by modulating macrophage subtypes. In this study, more M2 macrophages were produced in the STSr group compared to the ST group, while macrophage polarization, gene expression, VEGF and PDGF-BB levels all changed with increasing Sr^2+^ concentration. Furthermore, it was found that strontium-doped nanorod arrays had no significant promotion of HUVEC angiogenesis compared with pure sodium titanate nanorod arrays. However, STSr could enhance macrophage differentiation to the M2 phenotype and promote CD31hiEmcnhivesse formation. This suggests that Sr^2+^ might enhances the polarization of macrophages toward the M2 phenotype, which could promote vascularization and CD31hiEmcnhi vessel formation, a specific vessel subtype, strongly positive for CD31 and endomucin that couples angiogenesis and osteogenesis (Guo et al., 2020a). However, there is controversy regarding the mechanism by which Sr^2+^ promotes angiogenesis. A recent report suggests that Sr^2+^ may encourage the polarization of the N2 phenotype of neutrophils through the downregulation of the NF-kB pathway and increased STAT3 phosphorylation, promoting M2 macrophage switch and enhancing their inflammatory elimination function and ultimately promoting angiogenesis and tissue regeneration (Li et al., 2021). Therefore, more studies are needed to determine the pro-angiogenic mechanism of Sr^2+^ further.

### 4.3 Strontium Promotes Osteogenesis by Anti-Oxidative Stress Pathway

Reactive oxygen species (ROS) that are essential mediators in biological systems are involved in physiological processes such as cellular signal transduction and the regulation of intracellular homeostasis in pathological states. The ROS level is maintained at 1–15 μm in the normal physiological state, however, exogenous and endogenous stimuli can lead to abnormal production of ROS up to 500 μm or more in the tissue microenvironment. Excessive ROS accumulation can lead to cellular damage, oxidative stress (OS) and inflammatory responses, even interfering with the tissue repair process resulting in disruption of the tissue repair process. On the one hand, excessive ROS accumulation can disrupt tissue repair by causing oxidative stress and interfering with signaling pathways, on the other hand, excessive ROS can lead to DNA/RNA damage and protein dysfunction thus interfering with the normal immune response process (Shafiq et al., 2021). The presence of high levels of ROS in MSCs promotes lipogenic differentiation of MSCs and inhibits osteogenic differentiation by interfering with osteogenic signaling pathways (Atashi et al., 2015). It has been reported that ROS can participate in the RANKL/RANK signaling pathway as a second messenger, thus promoting osteoclast differentiation and maturation. At the same time, osteoclasts can produce large amounts of ROS via NADPH oxidase, which has a synergistic effect with MMPs and TRAP to destroy the bone matrix (Agidigbi and Kim, 2019). In addition, it has been shown that osteoblasts seem to perform their normal physiological functions only with strict removal of intracellular ROS (Cerqueni et al., 2021). Therefore, there is also a growing interest in modulating ROS by implanting new smart biomaterials to create an immune microenvironment conducive to bone repair.

Zhou et al. (2019) found that strontium-doped titanium significantly promoted osteogenic differentiation and inhibited lipogenic differentiation of MSCs in rats by reducing the production of ROS around the operative area. Related studies have shown that strontium can reduce intracellular ROS production by decreasing intracellular oxygen radical levels and increasing the expression of a range of antioxidants including superoxide dismutase (SOD), catalase (CAT) and glutathione peroxidase (GPx), producing an effect that promotes osteogenic differentiation (Jebahi et al., 2013; Zhou et al., 2019). In addition, Qi et al. (2016) further demonstrated the anti-oxidative stress mechanism of Sr by fructose strontium fructose 1,6-diphosphate (FDP-Sr) and found that strontium could inhibit ROS production by inducing the expression of the caspase 3, which could reduce the apoptosis of osteoblasts. Through simulating a microenvironment with high levels of OS, Shen et al. used samples with different levels of strontium doping (25%, 75%, 100%) to investigate how strontium affects bone regeneration by regulating ROS production and found that high Sr-doped samples (especially Sr100%) had positive effects on osteoimmunomodulation under the OS microenvironment. This may be related to the fact that a high dose of Sr2+ effectively eliminates the excessive accumulation of endogenous ROS in osteoblasts by enhancing the activity of CAT and SOD in the body; meanwhile, Sr2+ inhibits ROS production and alleviates the level of inflammation to affect the cytoarchitecture and CAT/SOD activity of macrophages, which promotes the polarization of M0 macrophages to M2 (Shen et al., 2022). Therefore, further research on the anti-oxidative stress mechanism of strontium-doped biomaterials and the use of new smart strontium-doped biomaterials to regulate OS in the body may be one of the strategies to promote bone repair.

## 5 Strontium-Doped Biomaterials

### 5.1 Bioceramics

#### 5.1.1 Strontium-Doped Bioactive Glass

Research on the bioactive glass (BG) as a class of synthetic inorganic biomaterials introduced in the early 1970s by Larry Hench has gone through three generations, from the first generation of molten bioactive glass represented by 45S5, the second generation of sol-gel bioactive glass (SBG) and the third generation of micro and nano bioactive glass (MNBG) developed in recent years. MNBG has a micro-nano structure and higher surface activity, which gives it outstanding advantages in biomineralization, osteogenic differentiation induction and bone tissue repair, therefore, MNBG is expected to be applied in bone repair better (Perez et al., 2014; Tsigkou et al., 2014). BG has excellent osteoconductivity and osteoinduction properties, inducing osteoblast differentiation and subsequent bone formation. To further improve the bioactivity of biomaterials, Sr^2+^-doped micro-nano bioactive glass has emerged, in which Sr^2+^ can play a synergistic effect together with other bioactive elements such as silicon (Si), zinc (Zn), calcium (Ca) released by BG, and reduce the release of pro-inflammatory cytokines (Wu et al., 2014). After contact with body fluids, Sr^2+^ dissolution from BG can be released quickly to form a layer of hydroxycarbonate apatite (HA) on the surface of BG, which can form a strong bond between BG and bone tissue. Sr^2+^ released from BG can also stimulate the expression of genes, including extracellular matrix components, osteogenic transcription factors and growth factors to promote osteogenesis, which makes it an ideal material for bone regeneration (Christodoulou et al., 2006; Jell and Stevens, 2006). Sr^2+^ also could inhibit the production and ability to bone resorption of osteoclasts resulting in better therapeutic effects. Naruphontjirakul et al. showed that strontium-modified bioactive glass has promising biocompatibility and bioactivity to promote osteoblast proliferation and improve its safety and usability (Naruphontjirakul et al., 2018). Zhang et al. prepared a novel injectable cement consisting of a chitosan-based binder phase and strontium-doped borate bioactive glass particles (designated Sr-BBG). Compared with a similar cement (BBG) composed of strontium-free chitosan-bonded borate bioactive glass particles, Sr-BBG cement had a better ability to promote osteogenic differentiation of hBMSCs: ALP activity and expression of Runx2, OCN, BMP-2, COL-1 and BSP were significantly increased in hBMSCs cultured on Sr-BBG cement. In addition, after 4 and 8 weeks of implantation in the *in vivo* critical size rabbit femoral condylar defect model, the bone volume at different distances around the material was compared by micro CT analysis, and it was found that Sr-BBG bone cement had a better bone regenerative ability than BBG bone cement at the implant-bone interface (Zhang et al., 2015). Recently H. Autefage et al. designed a porous, Sr^2+^-releasing, bioactive glass-based scaffold (SrBG) and showed that the SrBG formed a near-perfect fusion with the contacted bone, forming a more desirable lamellar bone. This suggests that SrBG has excellent potential for guided bone regeneration and is expected to be a future modality for the treatment of complex clinical bone defects (Autefage et al., 2019). With the rise of the concept of osteoimmunology in recent years, a series of novel Sr^2+^-doped biomaterials have emerged to orient the immune response in a direction that favors bone healing by modulating the sequential polarization of macrophages. For example, Zhang et al. (2016a) proposed the immunomodulatory effect of Sr^2+^-Substituted Submicrometer Bioactive Glasses in their research report, which provides a new research direction for the further development of BG. Man Luo et al. prepared a novel bone immunomodulatory IFN-g/Sr-dropped bioactive glass (IFN-g/SrBG). This scaffold can initiate the immune-inflammatory response by releasing IFN-g to polarize macrophages towards pro-inflammatory M1 phenotype at the initial stage of implantation and then initiate the repair phase by releasing Sr^2+^ from SrBG to polarize macrophages towards anti-inflammatory M2 phenotype at a later stage, which enables greater promotion of mature bone formation in the region of bone defects (O'Brien et al., 2019; Luo et al., 2021). These biomaterials modulating sequential regulation macrophage polarization to promote bone healing show great potential for future bone defects treatment. However, although strontium-doped BG has good biocompatibility, the fracture toughness and bending strength of most of these materials are in the range of 0.5–1 MPa m1/2 and 40–60 MPa, respectively, which are significantly lower than the fracture toughness of 2–12 MPa m1/2 and bending strength of 50–150 MPa for cortical bone, therefore, these biomaterials need to be further improved in load-bearing performance (Hench, 1991; Montazerian and Zanotto, 2017).

#### 5.1.2 Strontium-Doped Calcium Phosphate Bioceramics

Calcium phosphate bioceramics (CaPs) are the most common and widely used bioceramics because of their excellent osteoinductive and osteoinductive capabilities. CaPs including hydroxyapatite (HA), tricalcium phosphate (β-TCP/α-TCP) and biphasic calcium phosphate (BCP) are widely used as implant surface coatings, cement components and scaffolds for clinical applications (Jeong et al., 2019). CaPs that have osteogenic and osteoinductive properties are able to dissolve in body fluids to degrade and ion release (Jeong et al., 2019). These properties positively impact biological activity in terms of cell adhesion, proliferation and new bone formation, resulting in biomaterials with excellent osteoinductive and osteoconductive capabilities. Usually, the degradation of HA occurs at the surface of osteoclasts and is essentially insoluble, however, the β-TCP degrades too rapidly because of the high solubility. BCP is a mixture of HA and β-TCP depending on different mixing ratios that can improve degradation and it has been reported that a 60:40 ratio of HA to TCP might provide an optimal formulation with respect to bone resorption and final degradation (Jelusic et al., 2017; Steffi et al., 2018). In addition, in order to enhance the osteogenic, angiogenic and antimicrobial properties of biomaterials, some drugs and biological agents can be incorporated into CaPs, but it was found that these bioactive components were only loaded on the surface of the material and showed a sudden release effect which had no long-term therapeutic effect for body, thus a way to incorporate trace elements into CaPs to obtain a more durable release and safe was created (Wan B. et al., 2020). Sr^2+^ is widely incorporated into CaPs due to its dual role of enhancing osteoblast activity and inhibiting osteoclast proliferation. It has been demonstrated that Sr^2+^ incorporates into HA components by replacing Ca^2+^ and plays a crucial role in the mineralization of bone (Demirel and Kaya, 2020). Pierantozzi et al. show a novel solvent-free extrusion-based 3D printing method that provides an easy way to co-print from the raw form of biomaterials. Composite scaffolds with polycaprolactone (PCL)-based polymer matrix, HA, and bioactive-enhanced phases of SrHA with Sr^2+^ concentrations of 0, 10, and 20 wt%, respectively, were synthesized by a practical single-step and solvent-free extrusion 3D printing technique. In in vitro assays, higher mineralization levels were observed for the SrHA-containing scaffolds compared to bare PCL and PCL/HA scaffolds (Pierantozzi et al., 2020). In recent research, Yan et al. prepared an interconnected porous microcarriers Sr10-HA-g-PBLG (10 mol% of Ca^2+^ in HA was replaced by Sr^2+^) by grafting poly (γ-benzyl-l-glutamic acid) (PBLG) on Sr-doped HA nano-particles. The Sr10-HA-PBLG microcarriers showed a long-term release of Sr^2+^ and a controlled degradation rate. Cellular evaluation with rabbit adipose-derived stem cells (ADSCs) shows that the material promoted the effects of cell infiltration, adhesion, proliferation and osteogenic differentiation. The ADSC-containing Sr10-HA-g-PBLG microcarriers showed more desirable bone mineralization and bone regeneration in rabbit femoral defects compared to ADSC-free microcarriers (Yan et al., 2018).

Recently, novel nanoscale bone cement has shown great potential for osteoporosis treatment due to its unique nanoscale morphology and physicochemical properties such as hardening ability, high surface area and protein loading capacity (Seo et al., 2021). In the past decades, two major systems have been formed during the development of biologic bone cement: Calcium phosphate cement (CPC) which shows good biocompatibility as well as polymethyl methacrylate (PMMA) bone cement which shows poor biocompatibility. Traditional polymer bone cement, such as PMMA and glass ionomer bone cement, are widely used in dentistry and orthopedics, but their application is limited by limitations such as insufficient osseointegration and poor mechanical properties. At present, it has become a research focus to use bone cement for osteoporosis therapy by improving bone cement with Sr^2+^-doping. Wu et al. developed an enhanced calcium phosphate hybrid cement (Sr-CPHC), which improved the physicochemical and biological properties of CPC through strontium. The compressive strength and initial setting time of Sr-CPHC were improved compared to CPC; the compressive strength of Sr-CPHC was increased from 11.21 to 45.52 MPa, as well as the initial setting time was extended from 2.2 to 20.7 min. Sr-CPHC with desirable biocompatibility promotes ALP activity, calcium nodule formation and expression of osteogenesis-related genes, showing superior osteogenic induction. Meanwhile, Sr-CPHC upregulated the expression of Angiopoietin-1 (Ang-1) and VEGF, promoting the migration and tube formation of human umbilical vein endothelial cells (HUVECs) *in vitro*. Sr-CPHC shows great potential in angiogenesis compared to CPC (Wu X. et al., 2021). Lee et al. prepared Sr-nanocement, which could release Sr^2+^, Ca^2+^ and silicate slowly under osteoporotic conditions. The bone healing process was accelerated significantly by the combined action of silicate, Sr^2+^ and Ca^2+^. Sr^2+^ and silicate which inhibit osteoclastogenesis by acting directly on osteoclasts or indirectly through osteoblasts slow down bone resorption events at the same time. The ions released through the implanted materials together orchestrate the balance of bone remodeling and regeneration, providing a new idea for the treatment of osteoporosis (Lee et al., 2021).

### 5.2 Polymers

The polymers used in BTE can be divided into natural polymers and synthetic polymers. Natural polymers are highly similar to natural extracellular matrix (ECM) and benefit from outstanding biocompatibility, cell adhesion, low immunological potential and gradual bioresorbability (Bonani et al., 2018; Rao et al., 2018). Natural polymers that have been used in BTE include collagen, fibrin, elastin and other natural polymers such as chitosan, alginate, silk. However, natural polymers exhibit poor mechanical properties, therefore they need to be blended with other polymers with better mechanical strength to mimic the bone tissue of the body. In contrast, synthetic polymers overcome these disadvantages and have excellent fatigue resistance and mechanical strength. In addition, the mechanical and physical properties of synthetic polymers are more reproducible and predictable, as they are synthesized under controlled conditions. Aliphatic polymers are the most commonly used synthetic polymers, including polylactic acid (PLA), polyglycolic acid (PGA), poly lactic co-glycolic acid (PLGA) and PCL. However, because of the potential side effects of biodegradation products and the poor biocompatibility of synthetic polymers, the combination of Sr2+ functionalization with polymers to fabricate hybrid materials for bone regeneration becomes a promising approach. Lourenco et al. developed an injectable Sr-hybrid system consisting of arginine-glycine-aspartic acid (RGD)-alginate hydrogels crosslinked with Sr^2+^ and reinforced by Sr^2+^-doped hydroxyapatite microspheres. The strontium composite group showed higher material degradation, cellular infiltration and more new bone formation were observed in the center of the bone defect compared to the Sr-free material, suggesting that the introduction of Sr^2+^ facilitated bone regeneration by improving the osteoinductive properties of RGD-alginate hydrogels (Lourenco et al., 2017). Müller et al. found that amorphous Sr^2+^-polyphosphate microparticles (“Sr-a-polyP-MP”) significantly upregulate the expression of the genes encoding for ALP and BMP-2, compared with amorphous calcium-polyphosphate (“Ca-a-polyP-MP”). The implantation of Sr-a-polyP-MP in polylactic acid-hydroxyacetic acid copolymer (PLGA) microspheres accelerated the healing of critical size calvarial defects in rats (Müller et al., 2017). Silk fibroin is considered as a promising biomaterial for its excellent bioactivity and mechanical properties. Ma et al. developed a novel chondroitin sulfate/silk fibroin (SrCS/SF) hybrid membrane containing a microporous structure by combining chondroitin sulfate, strontium and silk fibroin, and evaluated the effect of this biomaterial on osteoblast and osteoclast production through macrophage immunomodulation. *In vitro* results showed that Sr^2+^ dose-dependently promoted bone markers related to osteoblast differentiation, bone formation and matrix mineralization, and decreased the expression of pro-inflammatory cytokines in RAW 264.7 cells. Furthermore, it was found that the expression of BMP2 and BMP6 in RAW 264.7 cells and Wnt10b was highest in the 10% SrCS/SF membrane group by comparing different strontium-doped concentrations, thus implying that 10% SrCS/SF membrane might contribute more to the regulation of the periosteal environment towards favoring anti-inflammation and osteogenesis (Fenbo et al., 2019).

### 5.3 Metal-Based Materials

As bone tissue regeneration materials, polymer and bioceramic scaffolds have received the most attention, however, there is much room to improve on their mechanical properties both in terms of strength and ductility. Titanium and tantalum are the most frequently used metal-based materials in medicine and dentistry for BTE due to their excellent mechanical strength, fatigue resistance and excellent biocompatibility (Hanawa, 2019). Surface chemistry alteration and nanoscale topographical modification by bioactive ions are crucial processes in the current design of the surface of titanium (Ti) bone implants. However, Ti cannot meet the requirements of rapid osseointegration during regenerative clinical applications, therefore surface modification has been applied to enhance its bone regenerative properties. Alloying of metallic materials with Sr^2+^ or Ca^2+^ can effectively adjust the mechanical and corrosion properties, as well as further promote the effect of bone regeneration (Glenske et al., 2018). Lee et al. (2016) studied titanium implants with bioactive ion surface modification by Sr^2+^ and demonstrates that a more desirable osseointegration effect was observed in Sr^2+^-modified Ti implants, which may be produced by the increased chemotactic capacity and osteoinductivity of MSCs through the induction of the M2 macrophage phenotype. Yu et al. designed Na_2_TiO_3_ nanorod with different amounts of Sr^2+^ doping, in which found that the introduction of Sr^2+^ upregulated the expression of osteogenic-related cytokines and showed strong osseointegration ability *in vivo* experiments. It suggests that the Sr^2+^-doped nanorod played a crucial role in immunomodulation and subsequent osseointegration induced by the implanted material (Yu et al., 2022). Li et al. modified the surface of porous titanium scaffolds with Sr^2+^ and Ag^+^ (abbreviated as AH-Sr-AgNPs) to achieve sequential release of Ag^+^ and Sr^2+^ to activate preosteoclast differentiation by manipulating macrophage polarization. Furthermore, it was found that 0.1 × 10^–3^ m of Sr^2+^ was effective in promoting the polarization of M2 macrophages compared to other concentrations and more pronounced osteogenic differentiation was observed after indirect co-culture of MC3T3-E1 with stimulated macrophages. *In vivo* experiments showed that AH-Sr-AgNPs were effective in stimulating bone formation around implants (Li D. et al., 2019). In addition Okuzu et al. incorporated Sr into the Ti surface by improved alkali and heat treatment methods to obtain the desired osseointegration earlier through the release of Sr^2+^. *In vitro* experiments by MC3T3-E1 cells showed enhanced expression of β-linked protein, integrin β1, cytokinin D1, osteogenic genes, and enhanced ALP activity and extracellular mineralization. *In vivo* experiments in rabbits showed significant bone implant contact and biomechanical strength of Sr-Ti compared to Sr-free counterpart (Okuzu et al., 2017).

## 6 Conclusion and Perspectives

Modifying implanted biomaterials by adding trace elements to improve the therapeutic effect is commonly used in BTE. Choosing the type and concentration of trace elements in biomaterials is one of the main challenges in the field of modern biological tissue engineering and regenerative medicine. In recent years, studies on the mechanism of Sr^2+^ promoting bone regeneration by inducing macrophage polarization have gradually increased. More and more domestic and foreign researchers have studied the mechanism of strontium at the molecular and cellular levels, but there are still different opinions on how Sr^2+^ promotes bone regeneration through the immune system. The optimal concentration of Sr^2+^ in bio-scaffolds is still controversial, and the selection of the appropriate dose of Sr^2+^ to promote bone regeneration has become an issue that must be addressed in future research.

BTE attempts to mimic different aspects of bone structure/function through bionics to achieve and support new and functional bone tissue growth. However, the current bionic content consists of static mimicking of bone tissue composition, structure, mechanical properties and bioactivity through biomaterials rather than dynamic mimicking of the three phases of the whole bone reconstruction process. In recent years, macrophage research has intensified with the rise of bone immunology. Immunomodulatory therapy is becoming an active area of research. Nevertheless, the cross-talk between the skeletal and immune systems during bone healing is frequently overlooked. Programming the dynamic changes in macrophage polarization is essential for managing inflammation in orthopedic implants and scaffolds. However, existing studies remain ambiguous regarding the optimal time to induce a phenotypic shift in macrophages. It remains a significant challenge to determine the optimal time to convert pro-inflammatory macrophages into anti-inflammatory macrophages. We propose a new idea: is it possible to combine the timing of macrophage polarization with the three phases of bone reconstruction mentioned above? According to the different environmental characteristics of the three phases of bone reconstruction, the M1 phenotype is induced during the inflammation phase through surface modification of the implanted biomaterial, and the induction of the M2 phenotype is initiated when entering the repair phase. We can display and deliver macrophage regulatory signals in a precise and near-physiological manner, in an effort to optimize skeletal repair and restore skeletal function to achieve a dual bionic effect in time and space. Future research on the relationship between Sr^2+^ and macrophages and their clinical relevance may improve our understanding of the role of strontium in the bone formation process, providing the theoretical basis of bone immunology for the future modification of biomaterials. The ultimate goal is to achieve a modulation between bone regeneration and the immune system to utilize biomaterials to treat bone defects and other diseases effectively. Taking full advantage of strontium in better promoting bone healing through macrophage polarization and promoting osteogenesis by developing Strontium-doped biomaterials with osteoimmunomodulatory functions appear to be a new strategy for designing the next generation of orthopedic replacements.
